# Regulation of cholinergic basal forebrain development, connectivity, and function by neurotrophin receptors

**DOI:** 10.1042/NS20180066

**Published:** 2019-02-04

**Authors:** Zoran Boskovic, Sonja Meier, Yunpeng Wang, Michael R. Milne, Tessa Onraet, Angelo Tedoldi, Elizabeth J. Coulson

**Affiliations:** 1 Queensland Brain Institute, The University of Queensland, Brisbane, Queensland, Australia; 2 Clem Jones Centre for Ageing Dementia Research, The University of Queensland, Brisbane, Queensland, Australia; 3 Faculty of Medicine, School of Biomedical Sciences, The University of Queensland, Brisbane, Queensland, Australia; 4 College of Forensic Science, Xi’an Jiaotong University, Shaanxi, China

**Keywords:** axon innervation, axon guidance, basal forebrain, cholinergic, neurotrophins, synaptic plasticity

## Abstract

Cholinergic basal forebrain (cBF) neurons are defined by their expression of the p75 neurotrophin receptor (p75^NTR^) and tropomyosin-related kinase (Trk) neurotrophin receptors in addition to cholinergic markers. It is known that the neurotrophins, particularly nerve growth factor (NGF), mediate cholinergic neuronal development and maintenance. However, the role of neurotrophin signalling in regulating adult cBF function is less clear, although in dementia, trophic signalling is reduced and p75^NTR^ mediates neurodegeneration of cBF neurons. Here we review the current understanding of how cBF neurons are regulated by neurotrophins which activate p75^NTR^ and TrkA, B or C to influence the critical role that these neurons play in normal cortical function, particularly higher order cognition. Specifically, we describe the current evidence that neurotrophins regulate the development of basal forebrain neurons and their role in maintaining and modifying mature basal forebrain synaptic and cortical microcircuit connectivity. Understanding the role neurotrophin signalling plays in regulating the precision of cholinergic connectivity will contribute to the understanding of normal cognitive processes and will likely provide additional ideas for designing improved therapies for the treatment of neurological disease in which cholinergic dysfunction has been demonstrated.

## Introduction

### Neurotrophin receptors in cholinergic basal forebrain neurons

One of the defining features of the cholinergic basal forebrain (cBF) neurons is their expression of the neurotrophin receptors, p75 neurotrophin receptor (p75^NTR^) and tropomyosin-related kinase A (TrkA), TrkB and TrkC, the receptors for nerve growth factor (NGF), brain-derived neurotrophic factor (BDNF), and neurotrophins 3 and 4 (NT3/4).

By binding to their cognate Trk and pan p75^NTR^ receptors, neurotrophins mediate an array of morphological effects in mature neurons, which have been extensively reviewed elsewhere [1,2–4]. In the peripheral nervous system and hippocampus, where they have been most studied, neurotrophins can modulate neuronal development including neurogenesis and differentiation [5], circuit specification [6,7], and neuronal life and death decisions including programmed cell death [4,8]. Classically, neurotrophins are secreted both constitutively and in response to neuronal activity [1], acting as target-derived factors for post-mitotic neurons, with a need for the activated ligand–Trk receptor complexes to be retrogradely transported to mediate cell survival [9–11]. However, they can also act in a cell-autonomous manner [10,12], and within defined cellular compartments. In particular, neurotrophins directly modify synaptic activity (such as long-term potentiation and depression, LTP and LTD respectively), as well as regulating the morphology of synapses and spines [13,14], and the growth and pruning of axons and dendrites [15,16]. Although it is widely assumed that the effects of neurotrophins on basal forebrain neurons mimic these effects, this is far from resolved. Nevertheless, these growth factors are strong candidates for controlling and maintaining cBF connectivity and therefore cognitive and behavioural outcomes mediated by these neurons.

There are numerous unresolved questions pertaining to neurotrophins in cBF neurons. Do the neurotrophins play roles in all of the aforementioned aspects of cBF development and function? What is the major adult role of the neurotrophin receptors in this cell population? What is the evolutionary advantage for mammals to co-express all of the neurotrophin receptors in most cells of each of the nuclei that make up the basal forebrain, given their apparent overlapping functions? We propose that the complexity of cholinergic neurotransmission requires precise mechanisms for organising the heterogeneous population of cBF axonal projections to different classes of downstream neuronal targets, and this should be considered when developing therapies for conditions in which cBF neurons degenerate or are dysfunctional. This review will present the current knowledge regarding the roles of the neurotrophin receptors in cBF neurons during development, adulthood and dementia and consequently their influence on cognition.

## Origin and development of basal forebrain neurons

**Figure 1:**
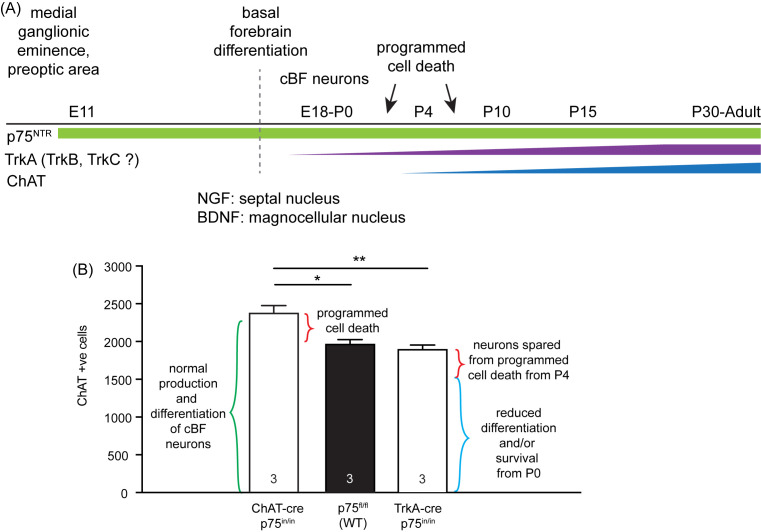
p75^NTR^ plays multiple roles in development (**A**) Timeline of mouse development indicating the expression of p75^NTR^, TrkA and ChAT. NGF and BDNF appear to play complementary roles in inducing the differentiation of septal and magnocellular cBF neurons, respectively. TrkA and TrkB also regulate axonal growth and ChAT expression but not cBF neuron survival. The age at which TrkB and TrkC expression is induced in cBF neurons is unclear. (**B**) Comparison of the number of cBF neurons in ‘wild-type’ (WT; p75^fl/fl^; black), and mice lacking p75^NTR^ from either E18 (via TrkA-cre; MGI:4360700; >95% recombination) or P4 (via ChAT-cre). ChAT-positive cells were counted in every third section (as described in [125]) in the MS, VDB and HDB of P30 animals. As both knockout strains lack p75^NTR^ expression during the postnatal period, which reduces programmed cell death (red bracket), the reduced cBF neuronal number in TrkA-cre p75^NTR^-deficient mice compared with ChAT-cre p75^NTR^-deficient animals indicates a role for p75^NTR^ in cBF neuronal survival or differentiation between E18 and P4 (blue bracket compared with green bracket). *n*=3 mice per genotype. *P<0.05, **P<0.01, one-way ANOVA.

An excellent resource regarding the development of the basal forebrain in different species is provided by Semba [17]. In the mouse, p75^NTR^ is expressed from embryonic day (E) 11 in the neuronal precursors of presumptive basal forebrain neurons (Figure 1). Expression of TrkA and markers of the cholinergic phenotype are found in the differentiating cBF neurons after birth, following their migration from the subventricular and marginal zones of the ventral telencephalon. TrkA is expressed from E18 to E20 [18] whereas ChAT and acetylcholine esterase expression are robustly detectable at approximately postnatal day (P) 4 in the medial septum (MS), vertical diagonal band of Broca (VDB) and preoptic area [17,19]. Their expression then significantly increases in the axons of cBF neurons, peaking at approximately P30 in mice.

Cortical interneurons and nearly all cholinergic neurons of the ventral forebrain originate from progenitor zones in the ventral telencephalon that are positive for the homoeobox transcription factor Nkx2.1. In the mouse telencephalon, Nkx2.1 expression starts at approximately E10.5 and is restricted to the medial ganglionic eminences (MGE) and preoptic area, zones mainly known as the origin of parvalbumin- and somatostatin-expressing interneurons that migrate tangentially into the cortex (reviewed in [20]). However, lineage tracing in Nkx2.1-positive progenitors has revealed that these cells also give rise to neurons in subcortical structures including the globus pallidus and the septal nucleus of the basal forebrain [21]. Interestingly, the expression of Nkx2.1 in the basal forebrain is retained into adulthood, in contrast with cortical interneurons, in which Nkx2.1 is rapidly down-regulated during their tangential migration [21,22]. Consistent with this, deletion of Nkx2.1 during embryonic development leads to almost complete ablation of both cholinergic and parvalbumin-positive neurons in the basal forebrain. Postnatal deletion of Nkx2.1 in cBF neurons decreases the expression of p75^NTR^ and results in a decline in cBF neuron number, indicating that Nkx2.1 regulates factors involved in the maturation and survival of these cells [22,23], such as Lhx8 and Islet-1 [24,25].

Although the ventral telencephalon (subpallium) is the birthplace of septal basal forebrain neurons (comprising the MS, the VDB and the horizontal diagonal band of Broca (HDB)), some reports suggest that a different progenitor population forms the magnocellular nucleus (that consists of the nucleus basalis of Meynert (NBM) and the substantia innominata (SI)), and that this population may originate from progenitor zones in the ventral pallium [17,26]. This is consistent with the observation that Nkx2.1-lineage derived neurons which co-stain for ChAT are found mainly in the MS and VDB, although not in the HDB [21,26]. Rather, cells that are negative for Nkx2.1 but positive for reelin expression (a marker not expressed by septal cBF neurons) migrate from the ventral pallium to the subpallium and differentiate into cholinergic neurons of the magnocellular nucleus [26]. Nonetheless, both populations express p75^NTR^ and all Trk receptors, indicating important functional roles for these receptors in both septal and magnocellular cBF neurons.

### Role of neurotrophins in basal forebrain development

Exactly how the expression of Trk and p75^NTR^ receptors is regulated during embryogenesis is unclear. Although it is not unusual for TrkB and TrkC receptors to be co-expressed by neurons, the additional co-expression of TrkA, as occurs in the basal forebrain, is unusual in the brain [27]. Furthermore, TrkA expression in cBF neurons is an evolutionarily recent development, being found in mammals but not birds [28], which potentially indicates a new functional specialisation of cBF neurons. Several reports have demonstrated that bone morphogenetic protein (BMP) 9 is transiently expressed in the septum during basal forebrain development and up-regulates the expression of p75^NTR^, ChAT and acetylcholine synthesis both *in vitro* and *in vivo* [29–31]. Furthermore, BMP9 up-regulates NGF, ciliary neurotrophic factor and serpin F1, creating a trophic environment for cBF neurons. Non-cholinergic, TrkA-negative septal basal forebrain neurons also express low levels of NGF, permitting both autocrine and paracrine roles for NGF in the development and/or maintenance of cBF neurons [18,30]. Indeed, many earlier reports concluded that endogenous NGF regulates the differentiation of cBF neurons [32,33].

In the magnocellular nuclei, ChAT expression is induced by P14, via a BDNF-induced, p75^NTR^- and Trk-dependent mechanism [29]. Interestingly, NGF cannot act as a substitute for BDNF, perhaps reflecting the different embryonic origins of magnocellular and septal cBF neurons [27]. In P14 rat cultures cBF neuronal survival was mediated by NGF, BDNF and NT-4 whereas NT-3 had no effect at this time point but induced cholinergic neuron differentiation in cultures from E18 rats [34]. Co-treatment with NGF and BDNF resulted in a small but consistent increase in the number of surviving ChAT-positive neurons, whereas sequential treatments demonstrated that most cholinergic cells respond to both these factors, indicating that these cells co-express TrkA and TrkB.

As p75^NTR^ and Trk receptors are expressed during the differentiation and/or specification of the cholinergic phenotype of developing cBF neurons, one might expect that mice in which individual neurotrophin receptor genes have been deleted would produce robust and obvious cBF phenotypes (see sections below). However, cBF neurons are unusual in co-expressing TrkA, B and/or C and have redundant mechanisms of development. Multiple trophic factors may act in concert to regulate the survival and growth of cBF neurons during the early postnatal critical period of development, with NGF being a particularly potent survival-promoting factor, and other cortically derived factors being more important for the growth, branching, and navigation of axons in the target regions [35]. Another possibility is that the methods used have not been sophisticated enough to detect the specialised functional changes each receptor modulates at particular time points.

## Adult cBF anatomy

**Figure 2:**
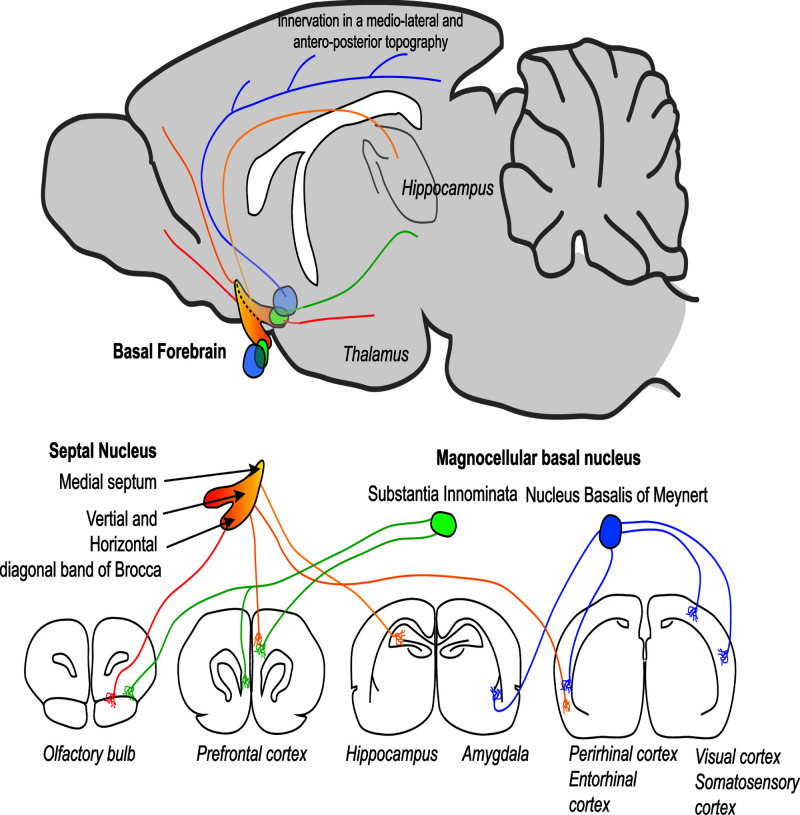
Anatomy and innervation of the basal forebrain nuclei Depiction of a midline sagittal section of the adult mouse brain illustrating the position of the basal forebrain nuclei (in ‘3D’) and their projections. Lower panels depict more precisely axonal innervation from specific basal forebrain nuclei to various brain areas portrayed in rostral to caudal coronal brain sections.

At completion of development, the basal forebrain consists of a heterogeneous cell population, including cholinergic, γ-aminobutyric acid-ergic (GABAergic) and glutamatergic neurons, which form complex networks that provide important modulation of cortical function [36,37]. As described above, the basal forebrain is subdivided into the septal nucleus and the basal magnocellular nucleus (Figure 2). In primates, the cholinergic nuclear groups are more commonly referred to as Ch1–Ch4 [38]. Ch1 (the MS) consists of ∼10% cholinergic neurons; Ch2 (VDB) has at least 70% cholinergic neurons, whereas only a minority (∼1%) of neurons in Ch3 (HDB) have been classified as cholinergic. Ch4, the basal magnocellular complex, is the least heterogeneous nucleus, with at least 90% of its neurons being cholinergic. However, some cholinergic neurons from the NBM can co-release GABA alongside acetylcholine, suggesting they perhaps have a different developmental origin [39].

MS/VDB neurons project to the hippocampus via the septohippocampal pathway (as visualised by magnetic resonance tractography imaging; [40]). These neurons simultaneously innervate parts of the prefrontal cortex [41], whereas HDB neurons project mainly to the olfactory bulb, but also to the thalamus. The NBM and SI send projections to the amygdala and innervate the entire neocortex, particularly the somatosensory barrel and visual cortices [38,42]. More specific features of the basal forebrain circuitry of the mouse have recently been described in detail [43,44]. However an emerging principle is that a single cholinergic neuron can project to multiple brain areas of associated function across cortical and subcortical areas [44].

The main afferent inputs into the basal forebrain are glutamatergic projections from the ventral tegmental area [45], GABAergic axons from the nucleus accumbens [46] and cholinergic projections from the pons [47]. There are also afferents from cortical, amygdaloid, striatal neurons. It remains unclear exactly which afferents from these regions contact cholinergic neurons in the basal forebrain, although noradrenergic and dopaminergic axons contact cholinergic neurons in extensive portions of the basal forebrain [48]. In addition, there is a largely unexplored network of intrinsic connections within the basal forebrain. It has been reported that a small population of peptidergic neurons [49] and parvalbumin-expressing GABAergic neurons [50] form synapses with cholinergic neurons within this region, and that the subpopulations of basal forebrain glutamatergic cells innervate local parvalbumin-expressing cells [51].

It is important to note that cBF axons can synapse on to specific neuronal targets (point to point neurotransmission) or evoke downstream responses via volume transmission, whereby the release of acetylcholine affects many neurons or synapses in the vicinity of the cBF terminal (see reference [43]). The presence of extensive axonal arbors and generic involvement of cholinergic control of theta oscillations across large brain areas has resulted in a misconception that there is little specificity in cBF axonal innervation [52,53]. More recent studies are challenging this idea, providing evidence that the connectivity of cholinergic projection neurons to their cortical targets must have sufficient neuronal and/or synaptic specificity to provide selective functionality.

A recent series of studies revealed several organising principles of cBF neurons [44,54,55]. Firstly, cholinergic projections to the neocortex are not diffuse, but instead are organised into segregated or overlapping pools of projection neurons along a rostral caudal axis. The extent of overlap between cBF neuronal populations projecting to the cortex depends on the degree of connectivity between the cortical targets of these populations (e.g. olfactory bulb, piriform and entorhinal cortices; Figure 2). Secondly, axons from very distinct cBF areas can innervate immediately adjacent cortical regions, and immediately adjacent cBF neurons can innervate disparate brain areas. These findings suggest that the connectivity of cholinergic projection neurons to their cortical targets has sufficient specificity to provide functionally and spatially selective signalling.

Consistent with the anatomically derived connectivity maps [43,47], recent functional MRI (fMRI) of humans has revealed that the network connectivity between the basal forebrain and other brain areas falls into three functionally distinct connectivity subdivisions. Each functional group is linked to a subdomain of the basal forebrain and has specific target regions, including within the hippocampus, insula, thalamus, and cingulate gyrus. However there are also regions of functional overlap, such as the salience network which is associated with all three cBF functional clusters [56]. Thus there is mounting evidence that the wiring of cBF neurons, although extensive, is guided by unknown underlying mechanisms which may turn out to be a key component of functional modulation of cognitive processes by these neurons.

## The function of adult cBF neurons

### cBF neuronal effects on behaviour

cBF neurons, through their release of acetylcholine, have been implicated in a wide range of cognitive processes and behaviours, including attention, fear-related processes and reward-mediated behaviour, addiction, memory updating, extinction and renewal [57–61]. Much of the early research that revealed the role of these neurons in the behavioural outcomes used lesioning methods, in which a saporin–conjugated p75^NTR^-specific antibody was used to create a lesion that was specific to cBF neurons [62]. Depending on whether the injection was made into the ventricle or into a specific nucleus, either entire basal forebrain complex lesions or MS/VDB- and NBM-specific lesions could be induced in a range of animals from rodents to primates. Rat cBF neurons were shown to be required for various cognitive tasks, including motor learning [63] and fear consolidation [59]. Lesions in mice resulted in deficits in working spatial memory in the T-maze [64] and poor utilisation of spatial cues in the Morris water maze [65–67] and Barnes maze [66], as well as impaired cue-independent (egocentric) navigation [68]. These lesion studies were complemented by pharmacological approaches in which cholinergic transmission was blocked with the muscarinic receptor antagonist scopolamine, which resulted in similar cognitive changes [69–73].

More recently, manipulating cBF neurotransmission in mice via optogenetics has further demonstrated cBF neuronal influences on cognitive processes. For example, selective activation of cBF neurons in different cBF nuclei has been reported to improve complex cortically dependent behaviours such as the encoding of natural scenes [74] and olfactory discrimination [75], maintaining wakefulness [76], and mediating cognitive functions through responses to external reinforcement feedback [77]. Not surprisingly, the cBF nuclei required for particular behaviours depend on the circuity involved in the task. MS/VDB cBF neurons are needed for hippocampal-dependent functions such as the detection of spatial cues, exploration and cue-dependent allocentric navigation [78], and prefrontal cortex-dependent fear extinction [79], whereas NBM cBF neuron-mediated neurotransmission is necessary for amygdala-dependent functions including fear conditioning (but not extinction), object recognition [59,80] and egocentric navigation (Hamlin, Boskovic and Coulson, unpublished).

### Mechanisms of cholinergic action on neurons and networks

Not all effects of the basal forebrain on behaviours and associative tasks require intact cBF circuits [68]. Rather, complex cognitive processing that requires the integration of information between multiple cortical regions appears to be specifically dependent on the appropriately timed release of acetylcholine from cBF neurons. In particular, it has been proposed that the basal forebrain is particularly important for regulating the output of cortical layer 3 neurons, responsible for invariant perceptual representation, as well as layer 5 pyramidal neurons that control memory output [81]. Layer 3 isocortical neurons are activated on association and recall of associative memories and project contralaterally to activate the layer 5 pyramidal neurons responsible for behavioural responses, which output to the cerebellum, pons, basal ganglia (specifically the subthalamic nucleus and striatum) and motor cortex.

Early work revealed that cBF-mediated neurotransmission also controls electrical oscillations in the hippocampus, as lesioning cBF neurons led to a decrease in low-frequency (6–12 Hz) theta oscillations [82]. These findings were further extended to show that denervation of the hippocampus via cBF axotomy affects the ability of animals to encode novel information i.e. form new memories within the hippocampus [83]. More recently, stimulation of cBF neurons in anaesthetized mice revealed that hippocampal theta synchronisation is mediated by an indirect pathway in which cholinergic neurons recruit and stimulate non-cholinergic neurons within the basal forebrain [84]. However, a direct septo-hippocampal cholinergic projection can alternatively increase the firing of hippocampal inhibitory interneurons with concomitant decreased firing of principal cells [84]. Activation of both cholinergic and non-cholinergic afferents causes a reduction in hippocampal pyramidal neuron firing and a more precise coupling to the theta oscillatory phase. These two anatomically and functionally distinct pathways appear to mediate information encoding, a requirement for memory formation. Furthermore, encoding of novel memories within the entorhinal cortex requires acetylcholine-mediated suppression of signalling at the interface of the CA3 and CA1 regions of the hippocampus [85,86]. Such changes in synaptic activity in the hippocampus are timing dependent [87] and require co-ordinated cholinergic modulation at both pre- and post-synaptic sites within this region [88].

Real‐time monitoring of acetylcholine release in the cortex of rats performing attentional tasks has revealed that precisely timed (transmission in the order of seconds; ‘transients’) cholinergic signals in the prefrontal cortex coincide with the detection of behaviourally significant cues [89], such as when animals are anticipating a meal [90], presented with sensory stimuli [91] or subjected to operant learning [92]. Cholinergic-mediated enhancement of post-synaptic activity, by optogenetic activation of cBF neurons, is not only associated with improved cue detection [93], but is also sufficient to condition cued-interval timing activity, thereby mimicking the reward timing activity that is observed after behavioural conditioning, and can improve visual discrimination [94]. Conversely, blocking cBF activity with optogenetics [92,93] or long temporal resolution cBF stimulation [95] synchronises activity across different cortical areas, thereby causing animals to ‘miss’ visual cues, and impairs visual responses.

cBF-mediated modulation of cortical activity also depends on the strength of concomitant inputs to the target neurons: cholinergic neurotransmission is inhibitory during quiescence, but excitatory when there is simultaneous post-synaptic activity [96]. By reducing the excitability of less active neurons and enhancing that of more active ones, cBF neurons increase the signal-to-noise ratio of incoming information. These results suggest that cBF neurons might facilitate memory formation by biasing the synaptic competition in favour of potentiated cells [97]. cBF neurons can respond to reward and punishment with high speed and precision [77]. Responses are scaled by the unexpectedness of reinforcement and are highly similar between the prefrontal and auditory cortices, which are innervated by the HDB and NBM nuclei, respectively [77]. This suggests that cBF neurons broadcast a rapid and precisely timed signal to enhance information integration [57,98,99].

Electrophysiological evidence points to the ability of the cBF-mediated cholinergic mechanisms to result in both (i) an enhanced probability of propagating cortical output in response to previously associated information, and alternatively, (ii) regulating new learning in response to salient or novel stimuli by releasing dendritic inhibition while suppressing low threshold pyramidal neuronal transmission [96]. Evidence of the ability of acetylcholine to enhance synaptic potentiation at single neuron resolution is provided by experiments involving stimulation of acetylcholine release, either by direct basal forebrain stimulation or via optogenetic activation of axons, leading to an enhanced response of glutamatergic neurons in the cortex, an effect which can last for more than 5 min [100–102]. However, this appears to be due to the ability of the cBF to drive the activity of a feed-forward inhibition/disinhibition microcircuit of inhibitory and excitatory neuronal subtypes within innervated regions, a mechanism that is conserved across the cortex [103,104]. Nonetheless, it has recently been reported that muscarinic activation of calcium channels within layer 5 pyramidal dendrites causes the generation of dendritic plateau potentials, powerfully transforming the pattern of dendritic integration of simultaneous input, leading to repetitive action potential output [102].

cBF neurons have axonal collaterals within all layers of a cortical column, but the modulation is layer specific, and can also vary according to cortical area and species. Examples are provided below and in Table 1 (for recent more comprehensive reviews, see references [105,106]). There is emerging evidence that cBF neurotransmission is particularly important for layer 1 interneurons which express nicotinic and muscarinic acetylcholine receptors (nAchRs and mAchRs, respectively [107,108]). Tonic (brief/resting) nAchR activation of layer 1 neurogliaform cells results in direct inhibition of layer 2/3 and 5 pyramidal dendrites [109,110]. However, phasic activation (high-quantity burst firing) causes silencing of the neurogliaform cells through M1 mAchRs, causing disinhibition of layer 2/3 apical dendrites [110]. The majority of layer 2/3 interneurons, including parvalbumin-positive basket and chandelier cells, do not express AchRs, with only ∼20% of layer 2/3 interneurons having depolarising nicotinic responses and ∼3% of interneurons becoming hyperpolarised in response to acetylcholine [107]. However, parvalbumin-positive interneurons can be inhibited by the activation of cholinergic-responsive layer 1 interneurons, which in turn results in somatic disinhibition of pyramidal neurons. Finally, despite sparse expression of nAChRs in layer 5/6 pyramidal neurons, robust mAChR expression is maintained predominantly at the soma [107,108], where the receptors act to suppress single synaptic responses, but facilitate synaptic responses evoked during stimulation trains [111,112]. However M1 mAChRs are also distributed on the extrasynaptic membrane of pyramidal cell dendrites and spines opposed to cholinergic varicosities indicative of volume release sites [113]. Activation of dendritic M1 receptors modulates of dendritic excitability of layer 5 pyramidal neocortical neurons and powerfully controls dendritic integrative operations and neuronal associative computations [102]. Similarly, the effect of acetylcholine through nicotinic and M1 or M2 mAChRs modifies the ratio between excitation and inhibition, switching layer 5 pyramidal neurons from tonic to burst firing, and enabling a potent and sustained enhancement of the neuronal synaptic response [114].

**Table 1:** Examples of cholinergic receptor activation in the cortex that modulate neuronal excitability and synaptic plasticity

Neuron affected	Cholinergic response	Direct effect	Downstream effects
Layer 1 inhibitory neuron (NGF expressing)	Tonic cholinergic transmission: activation of nChR-expressing cells	Direct inhibition of layer 2/3 and 5 pyramidal dendrites	Inhibition of PV-positive interneurons causing axo-somatic disinhibition of pyramidal neurons
	Phasic cholinergic transmission: silencing of recipient cells through M1 mAchRs	Disinhibition of layer 2/3 apical dendrites	
Pyramidal neurons (layers 2/3 and 5/6): (BDNF expressing)	Axo-somatic activation of M1 mAChRs at location	Suppression of single synaptic responses, but facilitation of synaptic responses evoked during stimulation trains	
	Dendritic activation of M1 mAChRs	Compartment-dependent modulation of dendritic excitability	Direct enhancement of dendritic integration and computations
Thalamic axons (layer 4) (NT3 expression?)	Activation of presynaptic nAChRs	Amplification of thalamic neurotransmission	Increased strength of incoming sensory information

Taken together, the above data indicate that cBF neuron-mediated neurotransmission requires temporally precise modulation of post-synaptic targets by these cells. There is also a requirement for a high degree of anatomical organisation of (i) cBF axonal innervation to dendrites, neurons and microcircuits within cortical columns, (ii) cBF neurons within the same basal forebrain nuclei innervating distinct cortical regions, and (iii) coincident transmission to downstream targets by cBF neurons located in different nuclei. It is as yet unclear how cBF neurons are able to establish and maintain such a dynamic range of connections. This also raises the question of whether directed modulation of post-synaptic targets by cBF neurons is achieved through a high degree of plasticity of their connections in the adult brain. We propose that neurotrophins provide a key mechanism that could provide a cBF-cortical neuron organisational structure within target areas. However, how the cBF neurons receive incoming information from cortical or subcortical relays in an organised way, and innervate the cortex in a rostral caudal manner are topics outside the scope of this review.

## The role of neurotrophin receptors in cBF function

The neurotrophin receptors are co-expressed to differing extents in developing cBF neurons [18,47,115] and their expression persists throughout adulthood (Figure 1A). Over 90% of cBF neurons express p75^NTR^ in both rodents and primates [116,117]. In the adult human posterior NBM cBF nucleus, 54% of cBF neurons express TrkA, 75% express TrkB and 58% express TrkC [116]. The proportions are similar in the adult rat (at least 45% for TrkA and 77% for TrkB; [117]). The state of knowledge of the role of neurotrophin receptor in cBF neurons is explained below.

## The role of p75^NTR^ in cBF neurons

### Effects of loss of p75^NTR^ expression on cBF morphology

The majority of studies report that genetic knockout of p75^NTR^ leads to an increase in the number of cBF neurons, a finding that is proposed to be due to a lack of developmentally regulated apoptosis [118–120]. This is consistent with the reported role of p75^NTR^ developmental programmed cell death in a range of neuronal types, particularly peripheral neurons [121,122]. This phenotype was once controversial, due to conflicting reports from the use of germline knockout animals on a variety of different and mixed background strains, some of which express a remnant transcript [119,123,124]. However, we recently confirmed a role for p75^NTR^ in mediating cBF neuronal death during the early postnatal period (but not in age-related decline in cBF cell number) based on a conditional knockout of p75^NTR^ from P4 in the mouse cBF, using ChAT-cre-mediated deletion [25]. Interestingly, conditional knockout of p75^NTR^ in cBF neurons at birth using a TrkA-cre reduced the number of cBF neurons (Figure 1B), revealing a role for p75^NTR^ in facilitating cholinergic differentiation or survival between E18 and P4, prior to its cell death role. The programmed cell death period of septal cBF neurons occurs from P1 to P15, with a peak at P1 and a second peak at P5, following the induction of ChAT expression [126]. The apparent multiple roles of p75^NTR^ in cBF neurons (Figure 1A,B) mirror the effects of p75^NTR^ in mouse dorsal root ganglia, where p75^NTR^ together with TrkA is trophic at E15–E18, but mediates programmed cell death at P2 [127]. Thus, p75^NTR^ may facilitate Trk-mediated trophic effects during axonal targeting and prior to, or even concurrent with, its role in pruning ‘excess’ cBF neurons.

The absence of p75^NTR^ also affects the size of cBF neurons, which is thought to reflect an increase in the metabolic need of an enlarged axonal arbor [35]. Indeed, an increased density of axonal innervation of the hippocampus, somatosensory and visual cortices and amygdala has been reported for p75^NTR^-deficient cBF neurons [118,120,124,125,128–130]. However, the increased cBF axonal density could also be due to the increased cBF neuronal number, rather than, or as well as, an increase in the size of individual neuronal arbors. Although this is difficult to accurately delineate, labelling of individual axonal arbors has revealed that the arbor size of MS/VDB cBF neurons innervating the hippocampus and prefrontal cortex is not substantially affected by the lack of p75^NTR^ cBF neuronal expression [79], suggesting that the previously reported increased innervation was due to the overlapping arbors of the additional surviving cBF neurons. Despite this, the authors reported a ten-fold increase in synaptic connectivity between cBF neurons and target cells in the prefrontal cortex, an outcome that could be reversed by re-expression of p75^NTR^ in adult cBF neurons [79], thereby confirming a role for p75^NTR^ in pruning adult cBF connections. By contrast, synaptic connections to the dorsal hippocampus were relatively rare, and septo-hippocampal synaptogenesis was unaffected by manipulation of p75^NTR^ expression [79]. These observations indicate that p75^NTR^ controls presynaptic mechanisms of cBF synapse formation. They also highlight the possibility that different mechanisms control axonal and synaptic growth, and/or control cBF innervation of the hippocampus and prefrontal cortex. One possible mechanism could be mediated by the differential expression of neurotrophic ligands in the cBF target cells, acting through cognate Trk receptors expressed by cBF neurons, as discussed in the next section.

### Effects of loss of p75^NTR^ expression on behaviour and synaptic function

One of the most consistent observations of p75^NTR^ mutants lacking exon III (p75^exonIII−/−^) and cholinergic-specific p75^NTR^ knockout mice are changes in behaviour in cognitively complex tasks, with little change in baseline behaviour. In comparison with littermate controls, mice lacking p75^NTR^ have generally been reported to have improved spatial learning and/or memory performance in an egocentric passive place avoidance task, as well as in cued, hippocampal-dependent Barnes and Morris water mazes. The mice also display changes in the amount of time spent in light–dark compartments, and reduced cued fear conditioning and fear extinction consolidation [79,124,125,128,131]. These behavioural phenotypes are consistent with altered cBF neuron function, being often the opposite phenotype to that of mice with cBF lesions. Indeed, most of the aforementioned studies concluded that increased cell number and/or cholinergic innervation is responsible for the behavioural change. In only one study was re-expression of p75^NTR^ in adult animals attempted in order to reverse the observed phenotype. The study demonstrated that acute p75^NTR^ signalling, rather than the increased number of neurons (a developmental phenotype), is important for cBF neuron synaptic innervation and fear extinction consolidation [79]. However, it remains unclear which cortical cell types have ectopic basal forebrain synaptic connectivity, and what effect (excitatory or inhibitory) the enhanced cholinergic innervation causes.

Juvenile (2–3 weeks old) p75^exon III−/−^ and p75^exon IV−/−^ mice have been shown to have reduced hippocampal LTD, without an accompanying change in LTP [132,133]. Interestingly, 3–6 months old p75^exon III−/−^ mice have been reported to have enhanced LTP in the hippocampus and amygdala [130,131]. In each case, the absence of p75^NTR^ promotes the persistence of transmission between neurons, albeit by different synaptic mechanisms. Increased complexity of the dendritic spines of CA1 hippocampal pyramidal neurons, changes to glutamatergic receptors, and increased cholinergic transferase activity in p75^exonIII−/−^ mice have also been demonstrated [124,131,133,134]. In addition, it has been reported that, in adult mice lacking p75^NTR^, the excitability and persistent firing of pyramidal neurons in layer 5 of the entorhinal cortex is unregulated due to a lack of inhibition [135]. As the expression of p75^NTR^ in the entorhinal cortex occurs in the innervating cBF axons, the role of p75^NTR^ could be to regulate cBF neuron structure or function thereby indirectly mediating the inhibition or activation of post-synaptic pyramidal cell activity.

Although these phenotypes are consistent with the increased cBF arborisation of cBF neurons in p75^NTR^-deficient mice, whether they are due to p75^NTR^ signalling in cBF neurons during development or in the adult is difficult to determine. Experiments carried out using brain slices of developing animals have provided evidence of post-synaptic p75^NTR^ expression (e.g [133]), but cannot rule out effects of systemic changes to hippocampal physiology due to altered cBF innervation or other p75^NTR^-dependent developmental/neurogenic ramifications [124,136–138], which may also be mediated by changes in cholinergic function [139]. Therefore, the physiological results should be interpreted with caution. Nonetheless, altered cBF innervation to cortical neurons in p75^NTR−/−^ mice is expected to result in altered cortical synaptic plasticity, with associated cognitive changes. However demonstration of this will be best interrogated using adult conditional p75^NTR^ knockout strains.

It remains unclear what ligands drive p75^NTR^ signalling in cBF neurons, and whether p75^NTR^ acts to facilitate Trk receptor signalling as well as acting independently to facilitate synaptic pruning, driven by the developmental stage or expression levels of co-receptors. The current dogma would suggest that proneurotrophins, acting together with the sortilin/SorCS family of co-receptors, is likely to be responsible for the pruning of synapses and axons via p75^NTR^ (as has been recently reported for a developmentally transient, p75^NTR^-positive, prefrontal cortex-projecting hippocampal neuronal population [140]). Although mice in which sortilin receptor members are conditionally knocked out in cBF neurons have not been analysed, sequestration of proNGF with specific antibodies causes mild impairment in cognitively taxing, distractor attention tasks without affecting baseline attention [141], suggestive of changes to cholinergic neurotransmission.

## The role of Trk receptors in the cBF

Neurotrophin-mediated Trk receptor signalling appears to regulate both the development and adult function of cBF neurons. However, as with the analysis of p75^NTR^-deficient mice, it has often been difficult to differentiate the adult role from the developmental functions. Despite current dogma, target-derived neurotrophins are not essential for the survival of cBF neurons [142], but rather regulate their morphology and the extent of their cholinergic phenotype.

### TrkA-NGF mediated regulation of cBF neurons

The first study of TrkA-deficient mice revealed dramatically reduced acetylcholine esterase-positive fibres in the hippocampus and somatosensory cortex of those animals which survived to adulthood [143]. A personal communication cited in the report (L.F. Kroner, unpublished) indicated that these mice displayed no change in cBF neuron number. More recently, analysis of conditional NGF- and TrkA-deficient animals in which the gene was removed from E10.5 in nestin-positive progenitors revealed a 30% reduction in the number of cBF neurons at P7 that was still evident at 20 months of age when compared with cre-negative controls [144]. This was concomitant with a decrease in cholinergic axonal and terminal innervation to the somatosensory cortex and hippocampus, as measured by ChAT activity. The authors concluded that the decrease in ChAT- (and p75^NTR^-) positive cell number was due to the increased developmental death of cholinergic cells observed at P7. Although it is unclear whether the innervation phenotype represents loss of axons/cells or merely down-regulation of ChAT/p75^NTR^ expression, the authors reported that the expression of ChAT in the remaining neurons and the density of the remaining cholinergic varicosities were unchanged, suggesting that there was no difference in the connectivity of the surviving neurons. Therefore it is possible that a subpopulation of neurons was affected by the loss of NGF-TrkA signalling. Surprisingly, these TrkA-deficient animals displayed normal function across a large number of assays of cognitive domains, which included attention performance, anxiety, exploration and working and spatial memory. The authors concluded that deficits in TrkA signalling are not sufficient to induce cognitive impairments in adult animals, despite the mice displaying a significant cellular phenotype.

However, contrasting findings were reported in a study published the same year which used a different conditional TrkA knockout mouse line, in which the gene was removed from E9.5 in dlx5/6-expressing telencephalon cells [145]. These TrkA-deficient mice displayed mild cognitive deficits, with impaired associative and working memory, as manifested through decreased memory in a Pavlovian fear-conditioning task, and decreased object discrimination in a novel object recognition task (but no change in spatial memory in the Morris water maze task) compared with control animals. The number and size of cBF neurons in P30 mice was unchanged, although a significant decrease in cholinergic innervation to the hippocampus and frontal cortex was observed, as measured by p75^NTR^ immunostaining in the postnatal period, as well as a decrease in total ChAT expression in the basal forebrain across all ages investigated from P14 to P180. It is again unclear from these experiments whether this result represents a loss of the axons themselves or only down-regulation of ChAT and p75^NTR^ expression. However the authors suggested that loss of NGF-TrkA signalling (demonstrated by reduced Akt phosphorylation) specifically decreased the level of ChAT (and p75^NTR^) synthesis within cells, and that this in turn was sufficient to affect cholinergic neurotransmission.

It is likely that the explanation of these contrasting findings is, at least partially, due to methodological issues. In adult rats, down-regulation of TrkA in the magnocellular nuclei had no effect on cBF neuron survival but decreased the density of cortical cholinergic axons and reduced their capacity to release acetylcholine, impairing attention of aged animals [146]. Similarly, TrkA down-regulation in mice impaired performance in attention tasks, an effect that could be rescued by simultaneously sequestering proNGF [141]. Therefore, a common phenotype, evident from neonatal development onwards, is represented by reduced axonal expression of ChAT, and/or reduced innervation of cBF axons to the cortex. The most parsimonious overall conclusion is that NGF and TrkA signalling controls the expression of ChAT within cBF neurons and their axons, and has only mild effects on neuronal specification and/or survival. The conclusion that the major influence of TrkA is on acetylcholine production and release is consistent with the function of NGF, as reported for mice with reduced NGF expression, and is the opposite of that displayed by p75^NTR^-deficient mice.

#### The role of NGF in cBF function

The first study of complete NGF knockout mice indicated that the production and survival of cBF neurons was unaffected (when compared with the obvious effects on the peripheral nervous system; [147]). However, reanalysis of the published images suggests a decrease in adult cBF neuron number. Indeed, heterozygous NGF^−/+^ mice show a reduced number and size of adult cBF neurons, as well as deficits in memory updating in a Morris water maze reversal learning task [148], a phenotype that is highly reminiscent of MS cBF-lesioned [149] and p75^exonIII−/−^ mice [136], but is not seen in TrkA-deficient mice (see above). Nestin-cre driven conditional NGF^−/−^ mice, have a 30% loss of cBF neurons, reduced ChAT expression in axons (although not as severe as that in TrkA^−/−^ mice), and a reduced number of nerve terminals in the hippocampus and frontal cortex [144]. Similarly, mice with impaired NGF signalling through the expression of NGF antibodies from early postnatal development display a progressive loss of ChAT-positive cells in the adult cBF, cell shrinkage and reduced ChAT expression, as well as poor learning and retention in an 8-arm radial maze [150]. Experiments in which anti-NGF or anti-TrkA antibodies were applied to the rat basal forebrain at P2 have also been shown to cause a decrease of ∼75% in cBF cell number at P9–P22 and a reduction in the size of the remaining cBF neurons [151,152]. However, the effects of NGF antibody infusion in the latter experiment were reversible, indicating that NGF-TrkA signalling is not an essential survival factor during neonatal cBF neuronal development and the programmed cell death period [151].

One explanation for this is that NGF signalling plays a different role in regulating synaptic activity in immature and mature cBF neurons. Cultured cBF neurons from E16/17 rats are dependent on NGF for survival between the second and fourth week *in vitro.* However, at earlier or later time points, they are largely resistant to NGF deprivation. The addition of BDNF, NT3, NT4/5 or glial-derived neurotrophic factor to cBF neurons, or co-culture with cortical neurons that enable synaptic innervation, confers resistance to NGF deprivation during this critical period [35]. During the same period, when sequestration of endogenous NGF caused no effect on LTP or LTD in P16–P18 neonatal rat visual cortex slices, application of exogenous NGF blocked high frequency-stimulated LTP. In contrast, at P30–P35, high-frequency stimulation did not evoke LTP unless NGF was sequestered or muscarine was applied to the slices, indicating that the NGF effect was mediated by cBF neurons [153]. Therefore, NGF appears to act first to promote differentiation (via p75^NTR^?) and survival during maturation, then primarily as a mediator of postnatal axonal growth, and subsequently to fine-tune cortical synaptic function.

Several studies have directly tested the role of NGF signalling in adult cBF neurons, revealing that NGF can control synaptic plasticity, circuit development and rewiring. Intraventricular infusion of NGF into adult NGF^−/+^ mice was not only able to reverse the learning deficit induced by chronic NGF reduction, but also to cause an increase in cBF cell size and ‘number’ in the adult, indicating that NGF-mediated signalling controls the adult cBF neuronal phenotype [148]. Similarly, lesion of septo-hippocampal projections via the fornix of adult cBF neurons resulted in cell shrinkage and loss of ChAT expression, which could be restored by application of NGF [154]. NGF expression can also modulate adult cBF connectivity, and has been reported to augment plasticity and behavioural learning through cholinergic-dependent mechanisms [155–158]. Stimulation of NGF release through learning or transgenic expression has also been shown to attract and rewire cBF axonal collaterals from multiple cBF cell bodies, facilitating the recall of learned memories [159,160]. Moreover, it has been reported that topical application of NGF to the barrel cortex of an adult rat augmented the size of the functional representation of a whisker within minutes, replicating the effect of nicotinic stimulation [156]. NGF application also results in an increase in the amplitude of the activity-dependent intrinsic signal in the barrel of a stimulated whisker [155]. The timescale of this result indicates a role for NGF-TrkA signalling in regulating neurotransmission and/or synaptic plasticity rather than (or in addition to) structural change of the axon.

#### NGF expression is limited to interneurons

A particularly important detail is the target-derived source of NGF (secreted as proNGF; [161]) that cBF neurons encounter, with inhibitory interneurons emerging as the primary source of neuronal NGF. In the cortex, >90% of NGF-labelled cells are interneurons, with less than 3% of pyramidal neurons expressing *NGF* mRNA [162]. In the hippocampus, ∼70% of *NGF* mRNA-positive neurons are parvalbumin-positive, although ∼20% of parvalbumin-expressing interneurons do not express NGF. In contrast, only a subset of calbindin- and calretinin-positive interneurons (24 and 23%, respectively) contain *NGF* mRNA [163]. Although two-thirds of NGF-positive cells co-express parvalbumin, many parvalbumin-positive cells do not co-express NGF, and only a minority of NGF-positive cells are calbindin-positive (29.1 ± 3.9%) [164].

It has been demonstrated that cholinergic-responsive layer 1 vasoactive intestinal peptide (VIP)- and/or somatostatin-expressing interneurons can disinhibit the layer 2/3 parvalbumin-positive interneurons, thereby regulating pyramidal neuron firing [165]. However, in an apparent paradox to the NGF expression in parvalbumin-positive interneurons, those in layer 2/3 do not express receptors for acetylcholine, with only ∼20% of layer 2/3 interneurons having depolarising nicotinic responses and ∼3% becoming hyperpolarised in response to acetylcholine [107]. Furthermore, virtually every parvalbumin-positive neuron innervated by TrkA-negative GABAergic basal forebrain neurons expresses *NGF* mRNA [162]. One explanation is that NGF released by parvalbumin-positive neurons regulates *en passant* cholinergic neurotransmission to that local area. Indeed there appears to be a link between cholinergic and non-cholinergic transmission in cortical microcircuits regulated by the basal forebrain. For example, we recently found that a specific change of p75^NTR^ expression in cBF neurons has a feedforward effect on the cortical synaptic connectivity of non-cBF neurons as well as cBF neurons [79]. Whether this dual reorganisation is typical of the cBF neuronal response to target-derived influences deserves further attention. Nonetheless, it is clear that NGF is an important mediator of cholinergic synaptic function.

### TrkB-BDNF/NT4-mediated regulation of cBF neurons

**Figure 3:**
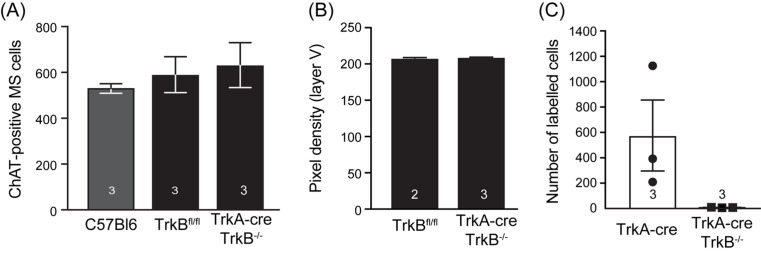
Postnatal loss of TrkB from cBF neurons affects synaptic connectivity (**A**) Quantification of the number of cBF neurons in the MS (defined as per [68]) of C57Bl6 mice, TrkB floxed (TrkB^fl/fl^) mice and animals lacking p75^NTR^ from E18 (via TrkA-cre). (**B**) Quantification of the cBF axonal innervation to the somatosensory cortex of TrkB floxed mice and TrkA-cre TrkB^−/−^ mice. (**C**) Quantification of the number of cells labelled by synaptic transfer of a herpes simplex virus expressing TdTomato from TrkA-cre expressing cBF neurons (methods are as described in reference [79]). No difference in cBF neuron number or innervation density was observed. However the number of prefrontal cortex cells labelled by cBF synaptic innervation in TrkA-cre TrkB^−/−^ mice was less than five per animal, compared with several hundred labelled cells in control TrkA-cre mice, the latter being equivalent to that reported for ChAT-cre animals [79]. The number of mice of each genotype is indicated in each graph.

Like NGF-deficient mice, complete BDNF heterozygous mice have fewer and smaller cBF neurons at P15, a phenotype that is maintained into adulthood. Although BDNF^−/+^ mice display decreased ChAT activity [166], there is also evidence that the reduced cell number is due to cell death, with increased DNA fragmentation in the MS at P6 being reported in these animals [167]. In contrast, we have preliminary evidence that conditional knockout of TrkB in cBF neurons from birth (using a TrkA-cre) does not influence adult septal cBF neuron number or the density of somatosensory cortex innervation to any layer (Figure 3A,B). This suggests that the reduction in BDNF expression throughout the brain during embryogenesis indirectly affects the postnatal survival of cBF neurons, rather than this being cell autonomous. If so, neither NGF nor BDNF is required for cBF neuronal survival during the postnatal programmed cell death period, although each may compensate for the other. Nonetheless, TrkB appears to be crucial for cBF synaptogenesis, as the number of synaptic connections made by TrkB^−/−^ cBF neurons to prefrontal cortical cells (labelled using an anterograde synaptic tracer; [79]) is significantly reduced so as to be almost negligible (Figure 3C), the opposite of the increase in synaptic connectivity observed in cBF-specific p75^NTR^-deficient animals [79].

In contrast with NGF, BDNF is expressed by pyramidal neurons and not interneurons and is therefore the best candidate for establishing pyramidal neuron cholinergic transmission via synaptic connections. Interestingly, topical application of BDNF has the opposite effect on the functional representation in the barrel cortex of a stimulated whisker to that of NGF application. BDNF results in a rapid and long-lasting decrease in whisker representation size and decreases the amplitude of the activity-dependent intrinsic signals [155]. This finding appears consistent with the actions of BDNF-expressing excitatory compared with NGF-expressing inhibitory neurons, although the explanation is unlikely to be that simple.

The effect of NGF, which mimics whisker training that causes the expansion of the receptive field for a specific whisker [155], may be due to a larger cholinergic neurotransmission release area within the cortical column (the area exposed to NGF) than is typically recruited by stimulation of whisker movement. In turn, the area which responds to cholinergic neurotransmission upon whisker stimulation (causing disinhibition/excitation) is larger. Surprisingly, this is not the effect of BDNF application, even though both NGF and BDNF are expected to enhance cBF axonal innervation, albeit to inhibitory and excitatory neurons, respectively. Rather, the effect of BDNF appears equivalent to denervation of a whisker, or alternatively, resulting in a more precise exclusive whisker representation field following whisker training [168]. That is, the effect of BDNF appears to be increased inhibition to (or reduced activation of) the application area. It is possible that this is because BDNF, unlike NGF, affects local cortical neurons (both inhibitory and excitatory) as well as cBF axons.

In summary, additional investigations need to be undertaken to reveal the mechanism by which BDNF-TrkB signalling modulates cBF neurons and thus cortical neuronal activity. BDNF appears to be required for the specification of magnocellular cBF neurons during embryonic development, and subsequently regulates the developmental and/or adult cBF synaptic innervation of the cortex, likely mirroring the role of NGF, but in relation to a different cellular target.

### TrkC-NT3 mediated regulation of cBF neurons

Finally, almost nothing has been reported regarding the role of NT3-TrkC in cBF neurons. It is known that NT3 plays a role in thalamic innervation of the cortex [169], where it is expressed in the rat from birth, but with expression in cortical layers varying during postnatal development [170]. NT3 may perform this role by modulating the extent of cBF innervation of the cortex, acting as a mediator of cholinergic control over afferent thalamic neurotransmission within the cortex. It could also control cBF innervation to specific cortical layers in order to establish the cholinergic regulation of thalamic or other afferent neurotransmission. In the adult, the expression of NT3 is much more limited than that of BDNF, which is expressed by all pyramidal neurons. Hippocampal cells expressing *NT-3* mRNA include those in the CA2 region, in the most medial portion of the CA1 region, and in the dentate gyrus [171]. These cells are primarily small subsets of parvalbumin- and calretinin-positive interneurons and form a distinct subset from the GABAergic cells that express NGF [162]. Surprisingly then, intraventricular infusion of NT3 is equally as potent as infusion of NGF in promoting cholinergic function and spatial maze learning of adult mice with lesioned cBF neurons [172]. This could be due to redundancy or overlapping functions, but given the expression of TrkC by cBF neurons, it is likely that the more restricted expression of NT3 controls aspects of cBF innervation to particular neurons or regions, which are not mediated sufficiently by TrkA and TrkB.

## Cholinergic degeneration in dementia

Almost 40 years old, the first major evidence-based hypothesis regarding the aetiology of Alzheimer’s disease (AD) was the cholinergic hypothesis (reviewed in reference [173]). It proposed that the depletion of brain acetylcholine in the cerebral cortex [174,175] and loss of cBF neurons, particularly in the NBM [176], was a primary cause of the cognitive decline which occurs in advanced age and AD. The loss is not specific for AD however, with cholinergic dysfunction and degeneration being associated with a range of other neurodegenerative conditions involving cognitive impairment, including Down’s syndrome, dementia with Lewy bodies and Parkinson’s disease with dementia [177–179].

In AD (the most prevalent and best studied dementia), it is now clear that atrophy of the basal forebrain (as measured by a reduced volume in MRI images) precedes and predicts clinical decline in cohorts with both mild cognitive impairment and idiopathic AD, as well as in individuals who have not yet been diagnosed with cognitive impairment [180–183]. As in animals with lesioned cBF neurons, only higher order cognitive functions such as spatial awareness and navigation are impaired in early dementia [184].

Furthermore, atrophy of the posterior NBM (Ch4p nuclei) has shown similar or superior diagnostic accuracy to hippocampal atrophy in identifying AD subjects [185,186], and recent work by others suggests that cBF degeneration precedes and, together with coincident amyloid peptide (Aβ) accumulation into plaques (the classic hallmark of AD), drives entorhinal cortical degeneration [187]. These findings are significant because adult loss of cBF neurons alone is sufficient to drive cognitive impairment and subsequent Aβ, but not tau, pathology in a range of transgenic mouse models of familial AD [149,188–192]. Conversely, we have recently demonstrated that deletion of p75^NTR^ from cBF neurons (resulting in more cBF neurons and more cholinergic synapses [79,125]) improves cognitive performance and reduces Aβ accumulation in the cortex of APP/PS1 familial AD mice [193]. These data strongly suggest that cholinergic degeneration is a major contributor to AD pathogenesis, whereas the maintenance of cholinergic function should be protective.

### Neurotrophins and AD

Neurotrophin dysfunction has been strongly linked to cholinergic degeneration in AD, particularly decreased NGF/TrkA trophic signalling [194,195]. This underpinned the rationale for neurotrophin-based treatments of this condition [196,197], including ongoing clinical trials delivering NGF to the NBM by implant or virus [198,199]. The specific neurotrophin-related changes which occur in AD include down-regulation of BDNF signalling, the accumulation of proNGF in the cortex, and reduced TrkA and TrkB neurotrophic signalling in cBF neurons in association with increased cholinergic dysfunction and cognitive decline (comprehensively reviewed in reference [161]).

p75^NTR^ small molecule modulators are also being tested for the treatment of AD [200]. Unlike in the healthy brain, there is evidence that p75^NTR^ expression is induced in the cortex and hippocampus of animal AD models and human patients [201–203]. Furthermore, neuronal dystrophy induced by Aβ in both cultured cells and mouse cBF neurons can be mediated by p75^NTR^ [204–206]. It has been shown that the extracellular domain of p75^NTR^ can bind to and sequester and/or endocytose Aβ [207–210]. Conversely, deletion of p75^NTR^ prevents the degeneration of cBF neurons in animal models of familial AD [204,206]. This evidence suggests that p75^NTR^ may be a key player in mediating the ongoing selective degeneration of cBF neurons, which in turn could promote not only cognitive decline but further Aβ accumulation and cortical degeneration.

The major question regarding neurotrophin-based therapeutic options is how best to prevent or restore the loss of cBF connectivity in order to preserve cognitive function. Is it sufficient to merely keep cBF neurons alive? The provision of NGF to the NBM appears to promote neuronal survival [199,211], but it is unclear whether the cBF axons remain connected to or reinnervate the cortex. The neurotrophic hypothesis suggests that the neurotrophin should be provided at the target. Whether an even distribution of axonal innervation across the cortex would suffice is unknown, although the evidence presented above regarding the precision of cholinergic innervation linked to function would suggest not. However, acetylcholineesterase inhibitors such as Aricept show mild to moderate efficacy even when the specificity of timing and/or neuronal response cannot be maintained by this class of drug [212,213]. Therefore, it remains to be demonstrated whether specific timing of and/or a precise location of axonal innervation and acetylcholine release in the cortex is required for efficacious neurotransmission, and whether neurotrophins or mimetics can be used to enhance cholinergic-based treatment avenues for dementia.

## Summary, future perspectives and predictions

cBF neurons are regulated by neurotrophins through their receptors from as early as cell specification and differentiation, mediating the extent of their cholinergic phenotype, and controlling axonal outgrowth and target innervation. In addition it is clear that the adult expression of neurotrophin receptors in cBF neurons regulates aspects of cell morphology, both growth and degeneration, which in turn can affect cortical function and thus behaviour. However, the precise role that each Trk receptor plays in these neurons is less clear, with redundancy being an unlikely explanation. Rather we propose that the neurotrophin receptors control specific aspects of cBF axonal innervation, and that the neurons remain in a developmental-like or plastic state throughout life, constantly rewiring in response to salient external stimuli to assist in encoding experiences required for future behaviourally relevant responses.

The specificity of innervation using different Trk receptors is illustrated by the sensory neurons of the dorsal root ganglia and neurons within the spinal cord [7]. These neurons also express p75^NTR^ and each Trk receptor (albeit in restricted subpopulations) during development, and depend on these receptors for their appropriate innervation of different targets based on the expression of target-derived neurotrophins. Furthermore, it has been proposed that neurons with overlapping axonal fields predominantly express TrkA receptors, whereas those neurons with restricted axonal fields predominantly express TrkB and/or TrkC receptors [27]. Extrapolating, this could suggest that NGF is responsible for volume transmission and BDNF for synaptic innervation. However, this model is too simplistic. No particular interneuron subpopulation or cortical layer consistently expresses NGF, indicating that only some cells or circuits have the ability to up-regulate cholinergic neurotransmission via NGF signalling, and there is no reported reason why all neurotrophins could not equally induce varicosities and *en passant* release sites. More akin to the peripheral nervous system, co-expression of multiple Trk receptors by a single neuron may ensure that all relevant cells receive innervation by cBF axons, to guarantee widespread coverage of the cortex. However, this too seems simplistic, given the apparent importance of the location and timing of cholinergic transmission for cortical function.

It has been proposed that the activity-dependent release of NGF could serve as a feedback signal between highly plastic cortical inhibitory microcircuits and cholinergic neurotransmission, whereby NGF acts not only to increase cholinergic activity but also to reorganise the circuits for cortical function [164]. In other words, NGF, acting through TrkA, promotes the cBF axonal innervation of the NGF-producing cortical targets, after which TrkA-mediated signalling tunes the degree of cholinergic neurotransmission by regulating the amount of acetylcholine that is produced and/or released. Alternatively, the up-regulation of ChAT by NGF may reflect the number or extent of axonal arbors or neurotransmitter release sites of individual neurons, which expand in response to an increase in the number of cortical cells producing NGF. In both examples, the expression of NGF is regulated by recently, robustly or repeatedly activated interneurons within particular microcircuits of the cortex.

**Figure 4:**
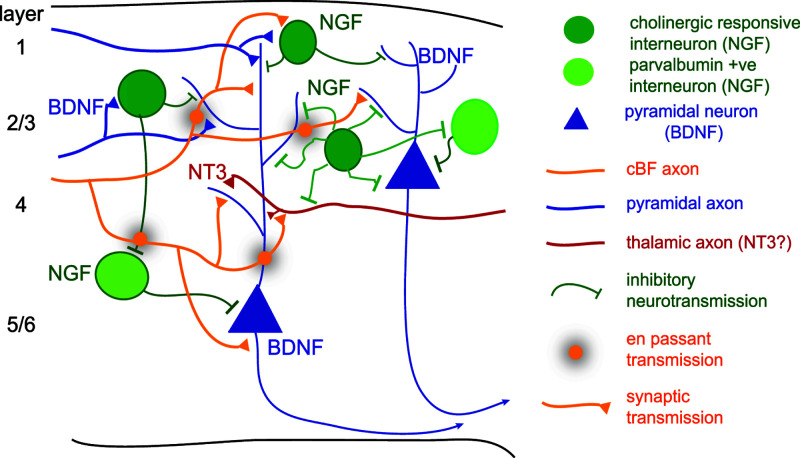
Model of cBF connectivity of the cortex driven by different neurotrophins cBF neuronal axons (orange) can release acetylcholine by both volume and synaptic neurotransmission. Some interneurons (green) express NGF and mediate inhibitory transmission, thereby regulating disinhibition. Other undefined interneuron subtypes do not express NGF whereas others do not respond to acetylcholine (e.g. parvalbumin-positive). Pyramidal neurons (blue) express BDNF and regulate cBF synaptic innervation of dendrites, to directly modulate dendritic input and scaling, as well as axo-somatic sites. The role of NT3 is as yet unclear but could modulate cBF regulation of thalamic axonal input (red), and/or control region specific innervation within the hippocampus (not shown).

An extension of this hypothesis would be that input requiring the encoding of new memories, which involves the rewiring of circuits, alters the NGF expression level or the neuronal cells that express NGF, to drive circuit or neurotransmission change, whereas recalling learned memory does not require NGF expression, or its expression remains unchanged. Furthermore, the release of proNGF by inactive interneurons, could stimulate pruning or LTD of local cBF connections via p75^NTR^. However, in this model (Figure 4) it remains unclear whether NGF only mediates cBF synaptic innervation of inhibitory neurons, or also regulates pyramidal neuronal activity through regulation of volume transmission within local cortical microcircuits.

As BDNF is only expressed by pyramidal neurons, and given our preliminary data from TrkB^−/−^ cBF neurons (Figure 3), it is reasonable to conclude that BDNF-TrkB acts to regulate the cBF synaptic connectivity of pyramidal neurons (Figure 4). The formation of cholinergic synapses onto pyramidal neuronal dendrites and their axo-somatic regions would help to co-ordinate dendritic integration and cortical synaptic output. However, BDNF can be released from axonal terminals as well as dendritic spines [214], and the release of BDNF from innervating glutamatergic axons could promote *en passant* cholinergic discharge and therefore modulate presynaptic nAChRs located at extra-synaptic sites on glutamatergic afferents [215], and thereby the strength of the incoming information as well as the integration within and the output of the information from cortical neurons.

NT3-TrkC is proposed to regulate other specialised functions of cBF neurons. Speculatively, this could include modulation of specific afferent neurotransmission, gatekeeping of information from brain areas (e.g. CA2/3 output to CA1), or control of innervation that needs to be coordinated between brain areas such as the hippocampus and cortex. However, NT3 may simply regulate a third layer of innervation such as a specific interneuronal class.

Finally, p75^NTR^ acts to limit cBF neurotransmission, which could be crucial when the information that cBF connections encode is no longer salient or requires modification. It appears that p75^NTR^ might act in a continuum, from inhibiting LTP and enhancing LTD at cholinergic synapses, to pruning cholinergic synapses and/or non-synaptic acetylcholine release sites, to pruning axons. It is possible that p75^NTR^ also acts to temper cholinergic transmission presynaptically, being able to regulate G-protein coupled inwardly rectifying channels that reduce the membrane potential, acting to prevent excitotoxicity in these highly metabolically active neurons [216,217]. Whether p75^NTR^ is activated by proneurotropins accumulating in the absence of sufficient circuit activity and cleavage to mature forms, or by other cell types that act to remove inactive synapses (e.g microglia), and/or in the absence of sufficient Trk receptor signalling, will require sophisticated *in vivo* experimentation to reveal. It is clear nonetheless that expression of p75^NTR^ makes cBF neurons susceptible to degeneration in conditions such as AD, including in response to Aβ.

Given the importance of acetylcholine on cortical disinhibition, particularly dendritic gating, further elucidating the mechanisms which control the cBF axonal innervation that results in synaptic and volume neurotransmission, as well as deciphering which neurons within cortical columns are so innervated, and under what conditions, will significantly increase our understanding of the cortical computations underpinning associative learning. Moreover determining the time-frame of cBF axonal and synaptic change mediated by neurotrophin receptors in the adult is of key importance for further understanding the mechanisms by which this influential group of neurons affects higher order cortical function and experience-dependent learning. This knowledge is also crucial for designing efficacious therapies to prevent or restore cBF dysfunction and thus cognitive impairment in the context of neurological and degenerative conditions including AD.

## Abbreviations

Abeta: amyloid beta
AD: Alzheimer’s disease
BDNF: brain-derived neurotrophic factor
BMP: bone morphogenetic protein
cBF: cholinergic basal forebrain
GABAergic: γ-aminobutyric acid-ergic
HDB: horizontal diagonal band of Broca
LTD: long-term depression
LTP: long-term potentiation
mAchR: muscarinic acetylcholine receptor
MS: medial septum
nAchR: nicotinic acetylcholine receptor
NBM: nucleus basalis of Meynert
NGF: nerve growth factor
NT3/4: neurotrophin 3 and 4
p75^NTR^: p75 neurotrophin receptor
SI: substantia innominata
Trk: tropomyosin-related kinase
VDB: vertical diagonal band of Broca
